# Degradation of Polylactic Acid Using Sub-Critical Water for Compost

**DOI:** 10.3390/polym12112434

**Published:** 2020-10-22

**Authors:** Toshiharu Goto, Mikitaka Kishita, Yin Sun, Takeshi Sako, Idzumi Okajima

**Affiliations:** 1 Innovation Exploring Dept., R&D Business Unit, Maxell, Ltd., Koizumi, Oyamazaki, Otokuni-gun, Kyoto 618-8525, Japan; gin.son.wp@maxell.co.jp; 2 Applied Chemistry and Biochemical Engineering Course, Department of Engineering, Graduate School of Integrated Science and Technology Shizuoka University, Hamamatsu 432-8561, Japan; kishita.mikitaka.16@shizuoka.ac.jp; 3 Energy System Section, Graduate School of Science and Technology, Shizuoka University, Hamamatsu 432-8561, Japan; sako.takeshi@shizuoka.ac.jp

**Keywords:** polylactic acid, decomposition, compost, sub-critical water

## Abstract

Polylactic acid (PLA) is expected to replace many general-purpose plastics, especially those used for food packaging and agricultural mulch. In composting, the degradation speed of PLA is affected by the molecular weight, crystallinity, and microbial activity. PLA with a molecular weight of less than 10,000 has been reported to have higher decomposition rates than those with higher molecular weight. However, PLA degradation generates water-soluble products, including lactic acid, that decrease the pH of soil or compost. As acidification of soil or compost affects farm products, their pH should be controlled. Therefore, a method for determining suitable reaction conditions to achieve ideal decomposition products is necessary. This study aimed to determine suitable reaction conditions for generating preprocessed PLA with a molecular weight lower than 10,000 without producing water-soluble contents. To this end, we investigated the degradation of PLA using sub-critical water. The molecular weight and ratio of water-soluble contents (WSCs) affecting the pH of preprocessed products were evaluated through kinetic analysis, and crystallinity was analyzed through differential scanning calorimetry. Preprocessed PLA was prepared under the determined ideal conditions, and its characteristics in soil were observed. The results showed that the crystallization rate increased with PLA decomposition but remained lower than 30%. In addition, the pH of compost mixed with 40% of preprocessed PLA could be controlled within pH 5.4–5.5 over 90 days. Overall, soil mixed with the preprocessed PLA prepared under the determined ideal conditions remains suitable for plant growth.

## 1. Introduction

There are at least 34,000,000 tons of plastic waste in the world, of which 93% is discharged into the natural environment through activities such as landfilling and ocean dumping [1]. As plastic waste is highly persistent and generates secondary pollutants, the demand for alternative materials that can reduce environmental loading has increased. This demand has accelerated the use of paper and biodegradable polymers. Paper is mostly made from cellulose, which is a common material in the natural environment, while a biodegradable polymer is an artificial material. It is important to ensure degradability before releasing a material into the environment. Polylactic acid (PLA) is the most widely used plastic variety of a biodegradable polymer, and it is expected to replace many general-purpose plastics for packaging and agricultural mulch [2,3,4]. However, some reports show that PLA disintegration is slow [5,6,7,8,9,10], meaning that PLA particles can remain in the soil for a considerable time. This implies the same problems as those associated with the use of general plastics. Therefore, recycling and pretreatment of PLA for composting have been extensively studied.

Recycling methods for PLA can be classified into processing and chemical recycling. In processing, PLA is recycled by remelting through fiber spinning of waste PLA [11,12] and the evaluation of PLA properties [13,14,15]. These methods are effective only for well-separated waste because contamination strongly affects the processability and properties of products. Thus, it is impossible to target all PLA products for recycling.

Chemical recycling has been investigated using processes such as alcholysis and hydrolysis. A previous study performed alcholysis using metal compounds and ionic liquids [16,17]. Disintegration of PLA using organometallic compounds has also been investigated [18,19,20,21,22,23,24]. However, these approaches are not suitable for compositing because of the use of chemical compounds, which add to the environmental load. Enzymes [25,26,27] and metal compounds [28] have also been used as catalysts for PLA degradation. Some environmentally friendly materials such as clay can be used, but they increase the treatment time by several hours, necessitating larger plants for degradation. From the perspective of industrial processing, chemical recycling generally requires a scale larger than that of composting or material recycling considering the scale of merit of chemical plants for disintegration and the application of recycled products. Therefore, in addition to chemical recycling, other methods are required for the treatment of PLA waste.

Composting is one of the attractive ways for waste PLA for the smaller scale distribution. However, some papers show that the biodegradation of PLA is very slow [29,30] in the soil. Another report indicates that PLA degradation does not occur or is very slow under 50 °C [5,6,7]. Soil temperature is lower than 35 °C [31], even in Vietnam, the tropics, which is lower than that of the composting procedure. This means that the decomposition of PLA in the natural environment requires more than a year. These results indicate that untreated PLA can be the source of microplastics in the future.

This issue has been investigated through various approaches. Several studies suggest the use of additives to accelerate PLA disintegration [32,33,34,35,36,37]. However, this strategy involves a trade-off between durability and degradability. Pantani et al. examined the decomposition rate during composting and the effect of crystallinity [38] and found that lower crystallinity results in faster degradation. In contrast, higher crystallinity decreases PLA transparency and increases heat resistance. Modification of the materials to ensure lower crystallinity may impose restrictions on product design. Consequently, the use of PLA would be limited.

Considering the abovementioned issues, a simple and fast pretreatment method for composting is required. Lunt [39] investigated the degradability of PLA in compost and found that micro-organisms tend to digest lactic acid oligomers with a number average molecular weight (Mn) of less than 10,000, producing carbon dioxide and water. In the above study, degraded PLA with an Mn of less than 10,000 could be obtained in 15 days of composting at 60 °C, following which decomposition was accelerated. Since molecules with Mn less than 10,000 decompose faster than larger molecules [39], the environmental load could be reduced by decreasing the Mn of PLA to less than 10,000 before composting or landfilling. Hydrolytic degradation has been reported to be an effective method for obtaining PLA monomers through decomposition [40].

We investigated hydrolytic degradation of PLA using sub-critical water to obtain compostable PLA oligomers, which are used as raw materials in lactide and copolymer production [41]. Sub-critical water is high temperature and high pressure water, with a temperature lower than the critical temperature of water (374 °C) and pressure higher than the saturated vapor pressure. Many properties of sub-critical water, such as the dielectric constant and ionic product, change continuously with changes in water temperature and pressure [42]. For example, the ion product of water is 10^−14^ mol^2^ m^−2^ at 25 °C and 10^−11^ mol^2^ m^−2^ at approximately 250 °C. Compared with liquid water at room temperature, sub-critical water dissociates into protons and hydroxide ions, which is expected to play a role as a catalyst. Furthermore, the dielectric constant of liquid water is approximately 80 at room temperature but decreases with increasing temperature. As a result, the affinity of water for the polymer increases, and hydrolysis of the polymer is expected to be accelerated. Several studies have reported decomposition using high temperature and high pressure water such as sub-critical water and supercritical water in chemical recycling of plastics; monomerization of PET [43], nylon 6 [44,45], and nylon 6,6 [46]; liquefaction of polyethylene [47] and polypropylene [48]; treatment of thermosetting resin such as epoxy resin [49] and phenol resin [50]; and treatment of fiber-reinforced plastic such as carbon fiber reinforced plastic (CFRP) [51] and GFRP [52]. PLA can be decomposed using sub-critical water, but it also hydrolyzes to the monomer depending on the reaction conditions. Therefore, in order to reduce the molecular weight of PLA while maintaining the solid state, it is necessary to determine appropriate reaction conditions.

Ideally, the production of acid should be restrained at the pretreatment because a large amount of organic acid in compost may contribute to the acidification of soil. Therefore, the following issues should be handled simultaneously:

1. Molecular weight reduction;
2. Restriction of production of small molecules, which are organic acids such as lactic acid and lactic acid dimer, because they contribute to soil acidification.

Kinetic analysis was performed to solve 1 and 2 above, and the range of conditions for PLA to decompose from low Mn solids to all water-soluble components was clarified. The range obtained here is the processing condition for obtaining solid low Mn PLA without producing organic acids. Based on these, we determined the optimum hydrothermal pretreatment conditions to obtain ideal products for compost. Many polymer blends using PLA have been reported [53,54,55,56,57], but we use pure PLA in this study to determine optimal conditions for controlling the degree of disintegration.

We evaluated the decomposition of PLA using the ratio of water-soluble components, which are represented by organic acids and the Mn value. The kinetics of Mn decrease and the production of water-soluble contents were analyzed using a first-order reaction, surface-reaction, and shrinking-core model [58] to verify the ideal condition for PLA degradation.

## 2. Materials and Methods

### 2.1. Materials

The PLA used in this study was Ingeo 3001D from NatureWorks Ltd., which is a grade of PLA commonly used in fiber and injection molding. Ingeo 3001 has a melt flow rate (MFR) of 22 g/min, 84,500 of Mn in terms of polystyrene, and 20.98% of crystallinity, and its clarity is transparent. PLA pellets were pulverized and used with a particle size of 1.0–1.4 mm. Distilled water was prepared using the ADVANTEC Water Distillation Apparatus (RFD240NA).

### 2.2. Batch-Type Reactor

**Figure 1:**
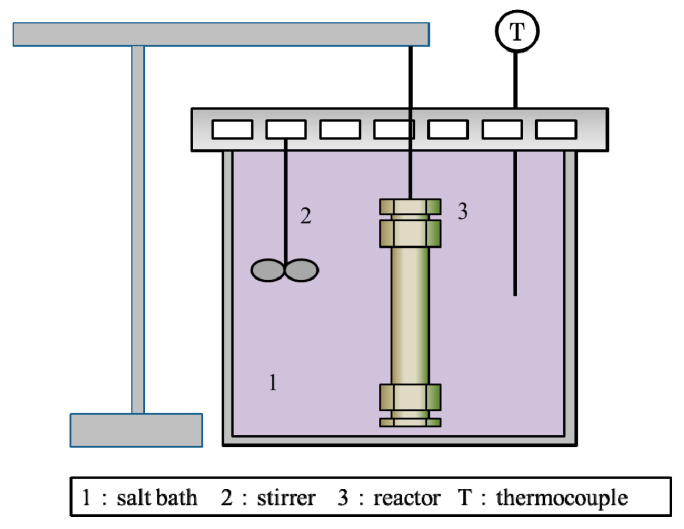
Apparatus for the decomposition of polylactic acid (PLA) using sub-critical water.

PLA was decomposed with sub-critical water, at temperatures of 180–250 °C and saturated water vapor pressures in a batch-type setup, as shown in Figure 1. The reactor consisted of a type-316 stainless steel tube with an inner volume of 8.9 cm^3^. Three experiments were performed, with sufficient water added to fill 75% of the reactor volume (5.85, 5.71, and 5.27 g, respectively) at reaction temperatures of 180, 200, and 250 °C, respectively. The reactor was filled with 75% liquid water to ensure that the chemical reaction progresses in the liquid state. The reaction pressure was set to the saturated water vapor pressure for the particular reaction temperature. The experimental condition is presented in Table 1.

In each experiment, predetermined weights of water and PLA were loaded into the reactor. The air in the reactor was not substituted with any other gas. The reactor was then sealed and heated in a salt bath for PLA decomposition. After the required time, the reactor was removed from the salt bath and cooled in room temperature water to terminate the reaction as quickly as possible. For safety, the reactor was cooled for 15 min, although only 1 min was required to cool the material in the reactor to room temperature. To recover the water-soluble products and residual resin from the reactor, water was added to the reactor, and the liquid and solid components were then separated by filtration. After drying in an oven for half a day at 60 °C, the residual solid PLA was accurately weighed using an electronic balance. The water-soluble content (WSC) of the PLA is defined by the equation.

WSC ratio of PLA (%) = [1 − Weight of residual solid (g)/Weight of the PLA (g)] × 100(1)

### 2.3. Analysis of yields

The Mn of residual PLA, which is essentially an index of decomposition of the main PLA chain, was measured using a gel permeation chromatography (GPC) system (HLC-8220GPC, made by TOSOH, Tokyo, Japan; column: SuperHM-M 6.0 mm I.D. × 15 cm, 3 μm, made by TOSOH) with two adjoined columns. A 0.2 wt % solution was prepared by dissolving the specimens in tri-chloromethane. Column and detection temperatures were set to 40 °C. The master curve, for the determination of Mn, was obtained from standard polystyrene. The heat of crystallization and the heat of fusion of decomposed PLA were measured using a differential scanning calorimetry (DSC) system (DSC60plus, made by SHIMADZU Corp., Kyoto, Japan; heating rate: 10 °C /min; reference: α-alumina; atmospheric gas: argon; and flow rate: 50 mL/min). Crystallinity was calculated from the following formula using these calories;

Crystallinity (%) = {[heat of crystallization (J/g) + heat of fusion (J/g)]/135 (J/g)} × 100(2)

where 135 J/g is the heat of fusion with 100% crystallinity of PLA [59]. The heat of crystallization and the heat of fusion are negative and positive, respectively.

Water-soluble materials were analyzed through high performance liquid chromatography (HPLC), using a Prominence system (controller: CBM-20A, pump: LC-20AD, and column: Shodex KC-811 300 mm × 8.0 mm) made by SHIMAZU Corp. The column oven and detector temperatures were set to 45 °C and 40 °C, respectively. Phosphating solution (pH = 2; 750 μm of phosphoric acid and 950 g of demineralized water) was used as the eluent.

### 2.4. Analysis of Kinetics

The decrease in *M*_n_ was analyzed as a first-order reaction. *Mn* at reaction time *t* is represented as follows:

Ln(*M*_n_) = −*k*_Mn_*t* + Ln(*M*_n0_), (3)

where *k*_Mn_ and *M*_n0_ are the kinetic constant and molecular weight at the reaction time of 0 min, respectively. Whether the reaction is well presented as a first-order reaction and the validity of obtained activation energy can be brought into question. The conditions required to obtain PLA with an *M*_n_ of less than 10,000 were calculated using the obtained Arrhenius equation.

The WSC ratio of PLA was analyzed as a surface reaction. The surface reaction and shrinking core model that is applied for the degradation of fiber-reinforced plastics [58] and other polymers was applied here to evaluate the induction time of the WSC ratio to obtain the water-soluble content.

### 2.5. Measurement of Soil pH

PLA was pulverized into particles of 0.5–2.8 mm and mixed evenly in the soil. For the soil, potting soil for home gardening (manufactured by Tachikawa Heiwa Nouen Co., Ltd., Kanuma-shi, Japan) was used. Only soil, 60 wt % soil + 40 wt % untreated PLA, or 60 wt % soil + 40 wt % decomposed PLA with sub-critical water (treated at 200 °C for 6 min with Mn = 10,000) were charged into flower pots with stones at the bottom. The ratio of PLA in the soil is extremely high in this study under the assumption that many preprocessed PLAs are present in the same soil. The pots were placed near the window in a room and illuminated with a growing light on cloudy days. Water was sprayed on all pots when the soil surface became dry. Soil temperature and pH were measured regularly; pH was measured using a soil pH meter (PH-212, manufactured by FUSO Co. Ltd., Tokyo, Japan).

## 3. Results

### 3.1. Degradation of PLA Chains

**Figure 2:**
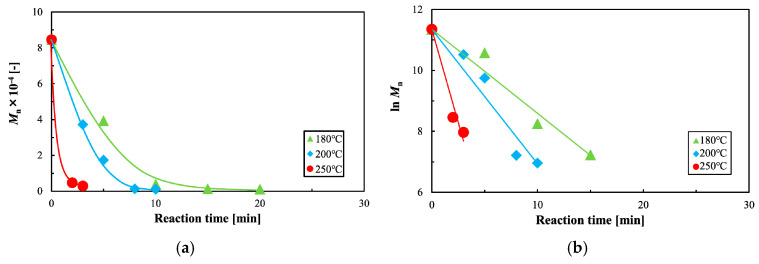
Kinetic analysis of polylactic acid (PLA) degradation. (**a**) Number average molecular weight (*M*_n_) of PLA after treatment with sub-critical water, according to reaction time and temperature and (**b**) analysis of the reduction in molecular weight due to sub-critical water, using a first-order equation.

Through GPC, the Mn of virgin PLA was determined as 84,500. The *M*_n_ of the decomposed PLA, which decreased with reaction time, is shown in Figure 2a. Moreover, a higher reaction temperature increased the rate of Mn decrease. This observed decomposition tendency is the same as that reported in previous studies [40], although the rate of decomposition was faster than reported. These differences in the decomposition rate are attributed to differences in the molecular weight, degree of crystallization, and the shape of PLA when loaded into the reactor. Further study is required to reveal the factors affecting the decomposition rate of PLA.

Figure 2b shows the results of the analysis of the rate of Mn decrease for decomposed PLA. The logarithm of Mn vs. the reaction time is well fitted to a linear equation, indicating that this reaction can be represented by a first-order equation. The kinetic constant *k*_Mn_ obtained by the least-squares method is shown in Table 2.

**Figure 3:**
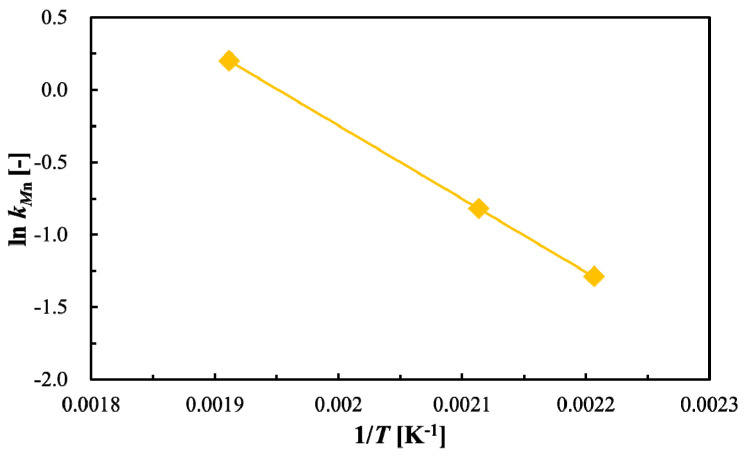
Arrhenius plot of reduction in polylactic acid (PLA) molecular weight in sub-critical water.

An Arrhenius plot of the kinetics constant is shown in Figure 3. According to calculations from the obtained gradient, the activation energy *Ea* and the pre-exponential factor are 41.95 kJ/mol and 1.887 × 10^4^ min^−1^, respectively. The obtained activation energy is also lower than the 49.6 kJ/mol value reported by Tsuji et al. [40]. This difference is also believed to be a result of differences in molecular weight, degree of crystallization, contents of catalysts for polymerization, and the shape of PLA loaded into the reactor. Further study is required to quantify the effects of these differences.

### 3.2. Crystallization of PLA

**Figure 4:**
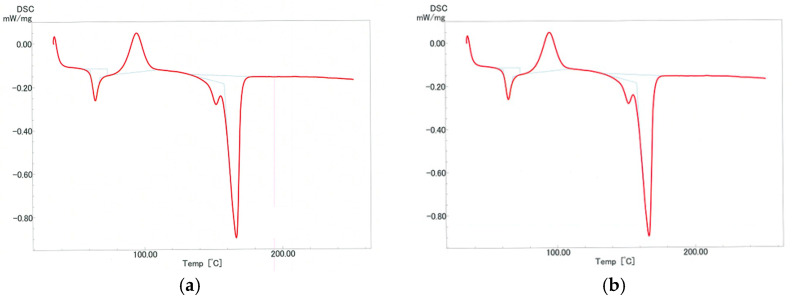
Differential scanning calorimetry (DSC) analysis of polylactic acid (PLA). (**a**) Untreated PLA and (**b**) decomposed PLA at 180 °C for 10 min.

**Figure 5:**
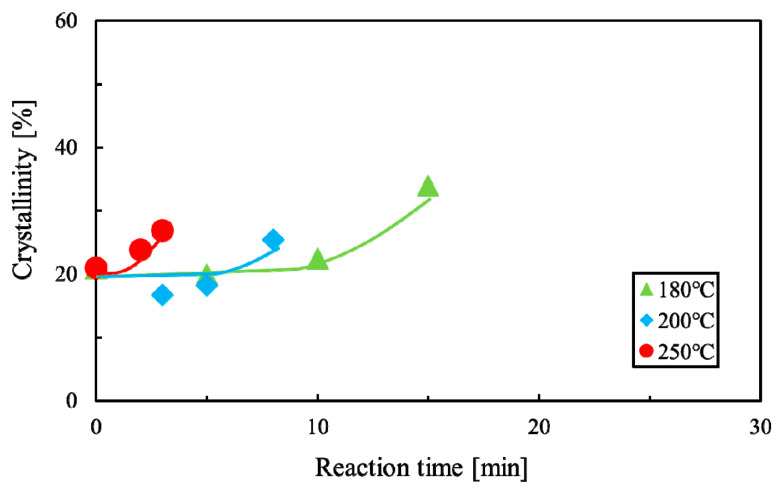
Dependences of reaction temperature and time of crystallinity of polylactic acid (PLA) after sub-critical water decomposition.

Figure 4 shows DSC thermograms of untreated and decomposed PLA. Table 3 shows the heat of crystallization and the heat of fusion of decomposed PLA. Using these values, the crystallinities were determined from Equation (2). The determined crystallinities are shown in Table 3. The dependences of the reaction temperature and time of crystallinity of PLA after sub-critical water decomposition are shown in Figure 5. Crystallinity was almost constant up to 10 min at 180 °C and up to 5 min at 200 °C, but increased with increasing reaction time. The crystallinity of PLA has been reported to increase in the early stage of hydrolytic degradation [60,61]. The same phenomenon was observed in the sub-critical water in this study. Although high crystallinity inhibits degradation, 30% crystallinity is not very high compared with that of molded PLA [62]. Therefore, these characteristics may not strongly contribute to delaying PLA decomposition in soil.

### 3.3. WSC Ratio of PLA

**Figure 6:**
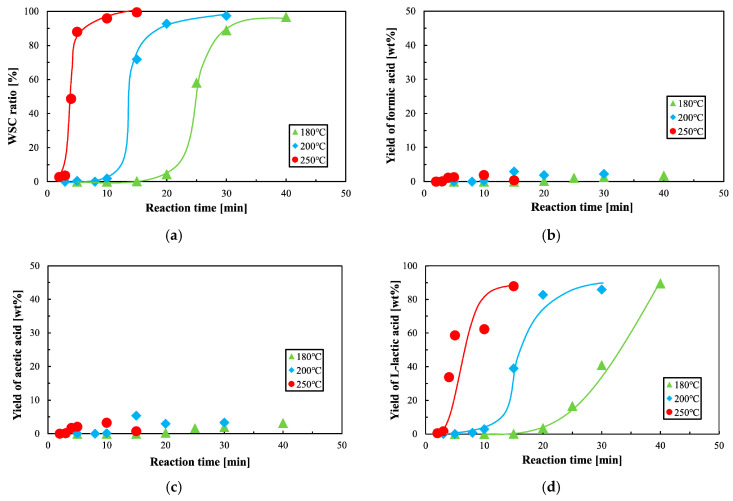
Kinetic analysis of the water-soluble content (WSC) yield. (**a**) WSC ratio of polylactic acid (PLA) in saturated water pressure; (**b**) yield of methanoic acid according to reaction time and temperature; (**c**) yield of acetic acid according to reaction time and temperature; and (**d**) yield of l-lactic acid according to reaction time and temperature.

The yield ratio of water-soluble components at different temperatures is plotted against reaction time in Figure 6a. The reaction time and induction time required for a certain yield decrease with increasing reaction temperatures. The yield of water-soluble components began after a few to 15 min of induction time, which is longer than the reaction time required to yield PLA with an *M*_n_ less of than 10,000, as shown in Figure 2a. These results suggest that there is an ideal reaction condition range for obtaining PLA with an *M*_n_ of less than 10,000, without yielding any water-soluble contents.

**Figure 7:**
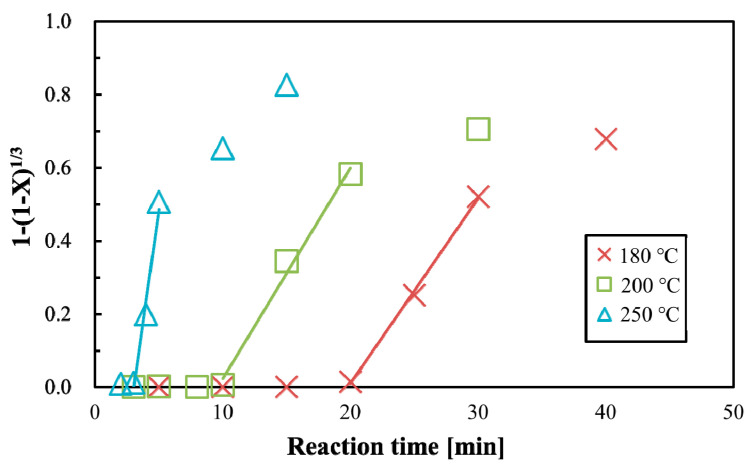
Analysis of water-soluble content (WSC) ratio of polylactic acid (PLA) in sub-critical water using the surface reaction and shrinking core model.

The surface reaction and shrinking core model [58] was applied because a sigmoidal curve was obtained for the WSC yield rate, as shown in Figure 6a. The kinetic constant *k*_l_ can be written as follows:

1 − (1 − *X*)^1/3^ = *k*_l_ (*t* − *t*_1_), (4)

where *X* and *t*_1_ are the ratio and induction time respectively and *k*_l_ is the kinetic constant of the WSC yield rate. The induction time was calculated using this equation and observational data. Figure 7 shows the kinetic analysis of the WSC yield rate of PLA using sub-critical water. The apparent rate constant *k_l_* was determined from the slope shown in Figure 7, obtained using the least-squares method. The obtained induction time and kinetic constant are shown in Table 4. The kinetic constant increased and the induction time decreased as the temperature increased.

### 3.4. Yield of Water-Soluble Components

The yields of formic acid and acetic acid are shown in Figure 6b,c. In our experiment, the production of these two types of acid increased very slightly with increasing temperature and reaction time. These acids may be the products of side reactions, caused by heat or oxygen, but the reactants are unknown. On the other hand, the yield of lactic acid, shown in Figure 6d, increased significantly with reaction time. This indicates that the hydrolysis reaction of the ester bond in PLA was occurring during the reaction. The yield of l-lactic acid was around 90%, in all decomposition conditions under 250 °C, which suggests that the side reactions were well suppressed. Previous experiments [40], in which replacement air was supplied, achieved a higher yield of l-lactic acid, indicating that side reactions can be controlled by replacing the air in the reactor.

#### 3.5. pH of Soil Mixed with PLA

**Figure 8:**
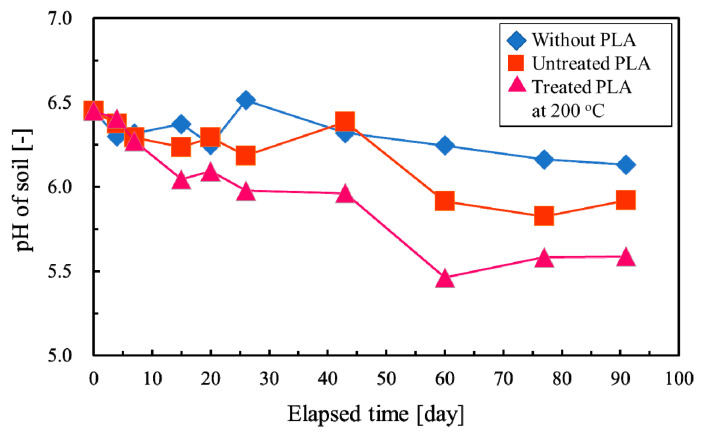
Effect of 40 wt % polylactic acid (PLA) addition on soil pH.

The effect of decomposed PLA with sub-critical water on soil pH was confirmed using two types of PLA: untreated PLA and decomposed PLA with sub-critical water at 200 °C. Figure 8 shows the effect of PLA addition on the pH of soil. As PLA was in powder form and mixed with soil, it was difficult to separate it from soil, and changes in the appearance of PLA could not be confirmed. Therefore, only the pH of soil was measured as an index of PLA decomposition and soil acidification. The soil temperature increased to 29 °C on the 15th day of measurement but remained between 20 and 25 °C at other times. The pH of soil without PLA addition was 6–6.5. In the soil mixed with untreated PLA, the pH did not change until 43 days after the start of measurement, after which it decreased to 5.8–5.9. The pH of the soil mixed with PLA decomposed in sub-critical water gradually decreased with elapsed time, and decreased to 5.4–5.5 after 60 days. Considering the unchanged pH of the soil without PLA, the production of organic acids such as lactic acid by the decomposition of PLA is presumed to decrease the soil pH. Furthermore, the pH of the soil mixed with sub-critical water-decomposed PLA decreased faster and reached lower values than that of the soil mixed with untreated PLA. These results suggest that PLA decomposed by sub-critical water is more likely to decompose in soil.

## 4. Discussion

This study aimed to establish ideal conditions for proper pretreatment of PLA for composting. A previous study [39] showed that for composting, the *M*_n_ of PLA should be less than 10,000.

The conditions for obtaining PLA with an Mn below 10,000 were predicted by the first-order reaction using Equation (3) as follows:

ln(10,000) > −*k*_Mn__‣_*t* + ln(84,500) (5)

The kinetics constant, obtained from the Arrhenius plot in Figure 3, is as follows:

*k*_Mn_ = exp(−5045.7/*T* + 9.8451) (6)

Substituting Equation (6) into Equation (5) results in the following equation:

*t* > −(ln(10,000) − ln(84,500))/exp(−5045.7/*T* + 9.8451) (7)

From Equation (7), the conditions for obtaining an Mn value of less than 10,000 can be shown in a *t*-*T* diagram.

Figure 6a–d shows that the WSC ratio changes with reaction time, and the yield of lactic, acetic, and methanoic acid increases. If these organic acids are added to compost, the fermentation could be inhibited by acidification of the compost. However, some release of organic acids may be beneficial for composting. The WSC ratio of PLA should therefore be controlled for compost. Figure 7 and Table 4 show the induction time of the WSC ratio, which is dependent on the reaction temperature. Consequently, induction time can also be plotted on a *t*-*T* diagram.

**Figure 9:**
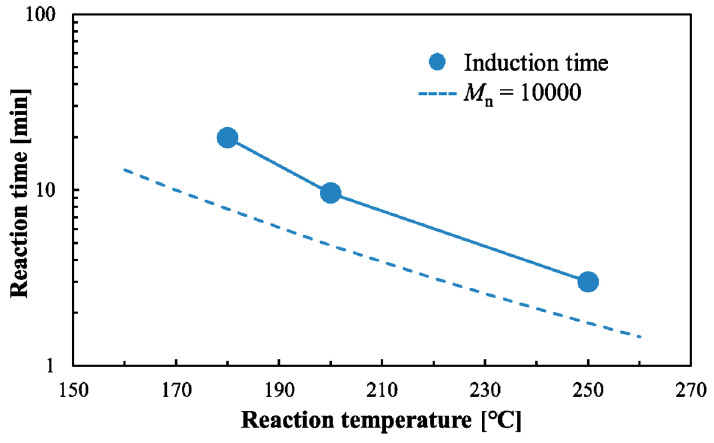
Induction time for the water-soluble content (WSC) ratio and reaction time for decreasing of *M*_n_ under *M*_n_ = 10,000 plotted against reaction temperature.

To evaluate the relationship between *M*_n_ and the WSC ratio on induction times, Equation (7) and the induction times obtained for different WSC ratios were plotted on a *t*-*T* diagram (Figure 9). The area between Eq (*t*) and the induction time shows the ideal reaction conditions for the pretreatment of PLA for composting using sub-critical water. This plot indicates that a higher temperature requires more precise control of the reaction time, to obtain solid PLA with a lower *M*_n_. This condition range is expected to depend on the type of PLA because the kinetics of decomposition is not precisely equal to those of the previous work [39]. Further study on expanding this range is needed to achieve stable production of solid preprocessed PLA with a low *M*_n_.

DSC analysis of preprocessed PLA shows that the crystallinity of products obtained under the proper reaction condition range is lower than 30%. Crystallinity, which suppresses PLA degradation, could be suitably controlled at values lower than that of molded PLA [62]. However, the history of heat strongly affects the crystallization rate of PLA [59]. Therefore, the history of heat in preprocessing should be controlled in the production process of recycled PLA. Moreover, composting with preprocessed PLA over a longer period should also be investigated. The pH of soil containing preprocessed PLA decreased faster than that of untreated PLA, indicating that the degradation of preprocessed PLA is faster under the proper reaction condition range than in soil with untreated PLA. The soil pH was maintained at 5.4–5.5 after 90 days even with 40% of preprocessed PLA under the condition range shown in Figure 9. The optimum pH range for many plants is between 5.5 and 7.5 [63], which means that soil of such high PLA content is expected to be suitable for growing plants. These results indicate that preprocessed PLA is usable as an addition to soil. Moreover, preprocessed PLA is expected to be used as a material for controlling the release of organic acids in soil to keep the pH around 5.5.

These results indicate that the proposed approach of using kinetic analysis and plots of reaction temperature and reaction time is effective for determining the reaction condition of PLA degradation for compost. This method is expected to be also applicable to other biodegradable polymers that produce organic acids such as PBS and PHBH.

## 5. Conclusions

The degradation of PLA by sub-critical water was investigated for composting. Kinetic analyses of decreases in molecular weight and WSC ratios show that these phenomena can be represented by a first-order reaction, and the combination of a surface reaction and shrinking core model equation, respectively. Kinetic constants and induction times were obtained. Using this approach, the ideal reaction condition range for obtaining PLA with an *M*_n_ under 10,000 and the point of WSC release were determined. The preprocessed PLA obtained under the determined ideal conditions was evaluated through DSC and soil pH. The DSC analysis showed that crystallization rate increased with PLA decomposition but remained lower than 30%, which is less than that of molded PLA. Crystallization by hydroxylation may not significantly suppress the degradation of PLA in soil. With the addition of preprocessed PLA, soil pH remained higher than 5.4–5.5 over 90 days even in soil containing 40% preprocessed PLA.

These results indicate that the kinetic analysis and the plot of the reaction temperature against the reaction time plot are useful for determining the reaction conditions of PLA degradation for compost. We believe that this research will help in efforts to reduce the environmental load of biodegradable plastics.

## Figures and Tables

**Table 1:** Experimental conditions.

Temperature (°C)	Pressure (MPa)	Density of Water (g/cm^3^)	Water (g)	PLA (g)
180	1.003	0.887	5.85	0.50
200	1.555	0.865	5.71	0.50
250	3.978	0.798	5.27	0.50

**Table 2:** Kinetic constant of decreasing number average molecular weight (*M*_n_).

Reaction Temp. (°C)	Kinetic Constant *k*_Mn_ (min^−1^)
180	0.2756
200	0.4404
250	1.2222

**Table 3:** Heat of crystallization and heat of fusion of polylactic acid (PLA).

Reaction Temp. (°C)	Reaction Time (min)	Heat of Crystallization (J/g)	Heat of Fusion (J/g)	Crystallization (%)
180	5	−15.84	42.74	19.93
180	10	−13.00	43.32	22.46
180	15	0	45.93	34.02
200	3	−22.19	44.80	16.75
200	5	−21.38	46.06	18.28
200	8	0	34.34	25.44
250	2	−11.82	43.97	23.81
250	3	−1.69	38.03	26.92
Before decomposition		−2.41	30.73	20.97

**Table 4:** Kinetic constant and induction time of the water-soluble content (WSC) ratio of polylactic acid (PLA).

Reaction Temp. (°C)	Kinetic Constant *k*_l_ (min^−1^)	Induction Time *t*_1_ (min)
180	0.0507	19.8
200	0.0576	9.5
250	0.247	3.0
